# Supplementary material for: Are carbon emissions trading and green financial instruments synergistic? -Comprehensive quantitative research based on content analysis
Source: PLoS One. 2024 Mar 7;19(3):e0298601. doi: 10.1371/journal.pone.0298601 (PMC10919601; doi:10.1371/journal.pone.0298601)
Supplement: S1 Data — (ZIP) [file pone.0298601.s001.zip › Data/地级市Z_F工作报告.zip!/╣π╢1⁄2/║╙╘┤╩╨/└┤╫╘╣1⁄2╓┌║┼í╛┬φ┐╦╩2╛▌═°í┐_2016.docx]

各位代表：

现在，我代表市人民政府，向各位代表报告政府工作，请予审议，并请市政协各位委员提出意见。

“十二五”时期工作回顾

“十二五”时期是我市发展史上很不平凡的五年。五年来，面对国际错综复杂形势、国内经济下行压力以及我市改革发展繁重任务，在省委、省政府和市委的正确领导下，在市人大及其常委会和市政协的监督支持下，市政府认真贯彻落实党的十八大和十八届三中、四中、五中全会精神和党中央、国务院的 一系列重大决策部署，深入学习贯彻习近平总书记系列重要讲话精神，主动适应新常态，抢抓发展新机遇，务实进取，攻坚克难，紧扣“三大抓手”，实施“四大战略”，大力开展“三赛”，努力建设“广东绿谷”，基本完成了“十二五”规划目标任务。

一、从发展看五年，主要指标实现翻番，综合实力迈上新台阶。2015年，全市生产总值810.08亿元，比上年（下同）增长8.1%，五年增长69.3%；人均生产总值26401元，增长7.5%，五年增长60.8%。全社会工业增加值416.28亿元，增长8.1%，五年增长105.7%；其中规上工业增加值380.32亿元，增长8.2%，五年增长112.6%。地方一般公共预算收入67.48亿元，增长11.6%，五年增长168.9%。固定资产投资564.14亿元，增长24.5%，五年增长178.2%。社会消费品零售总额482.99亿元，增长11.0%，五年增长88.9%。外贸进出口总额40.3亿美元，五年增长48.4%。城镇居民人均收入20016元，增长9.7%，五年增长51.9%；农村居民人均可支配收入10803元，增长9.3%，五年增长91.4%。

二、从产业看五年，项目与平台双提升，转型升级迈上新台阶。园区建设成效明显。市高新区成功升级国家级高新区，成为粤东西北地区首个国家级高新区。各县区成功创建省级园区，“优秀”园区占全省1/3。五年投入园区开发建设资金111亿元，扩园37平方公里；新签约项目569个，新投产项目358个，累计完成工业投资684亿元。共建园区取得实质性进展，投入对口帮扶资金13亿元。建成省级空气能检测中心、质量监督卫星导航产品检验站。截至2015年底，“两区六园”累计开发面积74.6平方公里，建成投产项目788个，其中规上企业420家，占全市79.2%。支柱产业集聚明显，电子信息、模具、硅产业等支柱产业不断发展壮大；其中电子信息产业产值575.06亿元、增加值90.75亿元, 五年分别增长2.7倍、2.1倍。园区工业总产值1203.38亿元、增加值295.11亿元、税收21.29亿元，五年分别增长180.2%、217.2%、151.2%。三次产业结构由“十一五”期末的13.3：48.0：38.7调整优化为11.5：45.8：42.7。民营企业实现工业增加值234.22亿元，增长12.1%，五年增长155.5%。“三大工程”成效明显。狠抓“四个一批”，大力实施“三大工程”，项目建设呈现生动局面。其中，“三个50”工程累计新签约项目383个、新开工项目335个、新投产项目301个；“三个100”工程累计新增规上工业企业218家、限上商贸企业371家；“双百亿”工程新签约26个、新开工15个10亿元以上项目。总投资100亿元的中兴通讯（河源）生产研发培训基地项目动工建设。创新驱动成效明显。自主创新能力不断提升，创建高新技术企业31家，新增省级工程技术研究中心14家、省级新型研发机构2个、省级孵化器1家。产学研合作进一步深化，与广东工业大学在市高新区共建了“河源广工大协同创新研究院”，引进了一批高层次人才，促进企业与高等院校、科研院所建立了战略合作关系。科技成果产出不断增多，2015年获省科学技术进步奖二等奖1项、三等奖4项，实现历史性突破。质量强市成效明显。产品、服务和工程质量稳步提升，五年新增省名牌（工业类）产品8个，标准化良好行为企业18家，制造业产品合格率稳定在90%以上；新开工建筑质量监督覆盖率100%，竣工工程质量全部达到国家标准或规范要求。生态旅游成效明显。启动建设万绿生态旅游产业园，竞得省扶持资金3亿元，吸引投资总额近200亿元的8个旅游重点项目落户。其中巴伐利亚养生庄园一期竣工开业，康泉18国际生态旅游城、客天下水晶温泉国际旅游度假区等项目顺利推进。新增国家4A级景区3家，建成国家、省休闲农业与乡村旅游示范县、镇、村13个。2015年接待入市旅客2536.81万人次，旅游总收入211.01亿元，五年年均增长35.6%。房地产、金融保险、商贸流通、中介信息、电子商务等取得长足发展。全市登记市场主体12万多户，五年增长71.7%。第三产业实现增加值345.65亿元，五年增长60.5%，年均增长9.9%。农业示范成效明显。灯塔盆地成功创建国家级现代农业示范区，核心区建设进展顺利。五年制定发布市级农业标准17项，新增省级农业标准化示范区10个，新增省级农业龙头企业21家、省名牌农产品11个，新增农民专业合作社3239家，辐射带动农户26万户，户均增收3100元以上。连平鹰嘴蜜桃被评为国家地理标志保护产品。完成农林牧渔业增加值95.92亿元，五年增长25.6%。

三、从城市看五年，提质扩容凸显格局，城乡建设迈上新台阶。城市扩容变化显著。坚持以规划为先导，编制了中心城区扩容提质建设规划、江东新区城市总体规划等一批城市规划，规划体系不断完善，规划指导性明显增强，土地节约集约利用水平提升。中心城区一体化进程加快，城市框架不断拉大，五年建成滨江大道等7条主干道，建成区面积达43.5平方公里、增加6.6平方公里。实施“跨江战略”，迎客大桥建成通车；江东新区建设开局良好，总投资106.69亿元的15个项目落户。县城框架进一步拉大，东源县城扩容50%、和平县城扩容40%、紫金县城扩容33%、龙川县城扩容32%、连平县城扩容15%。城市提质变化显著。改造提升沿江中路等24条市政道路，贯通文昌南路等11条“断头路”。大力改造提升旧城区，完成背街小巷改造工程100多项。对市区15处易涝路段进行整治。建成客家文化公园等7个公园，改造提升九重门公园等2个公园，建成全民健身广场等重点休闲广场，市区建成区绿化覆盖率提高到45%。新建和改扩建农贸市场12个。新建市商业中心、希尔顿酒店、万隆城等现代城市综合体，城市功能日趋完善。城市管理变化显著。大力整治城市市容市貌，顺利通过国家卫生城市复审，城市绿化、亮化、美化、净化水平显著提升，城市面貌焕然一新。建立健全土地长效管理机制，市区建设用地控规实现全覆盖。城市管理体制进一步理顺，城市综合管理执法和保洁水平明显提升。城市公交线路不断优化，公交覆盖面积250平方公里，五年增加200平方公里，公交分担率大幅提高。市区完成天然气置换。农村面貌变化显著。推进宜居村镇“六个一”工程，创建5个名镇、45个名村和18个广东省卫生村，完成村道硬底化2996公里。龙川县幸福村居示范县创建活动有序推进。农村综合改革不断深化，完成了农村集体资产清理核实工作。乡村金融服务站和银行卡助农取款服务点实现行政村全覆盖。完成农村集体土地所有权确权登记发证和农村土地承包经营权确权登记颁证试点工作。实行最严格的耕地保护制度，加快高标准农田建设，耕地总面积和基本农田保护区面积均超额完成省下达的目标任务，耕地总量连续五年实现占补平衡。

四、从交通看五年，区位条件日臻完善，基础设施迈上新台阶。交通条件提档升级。五年交通建设投入255.7亿元，其中2015年超百亿元。进一步完善以高速公路为主骨架、国省道干线为重要支撑、县乡公路为联络支线的交通运输网络，交通区位条件明显改善。高速公路成网，境内汕湛、大广高速公路建成通车，高速公路里程增加145公里，融入珠三角“2小时生活圈”，实现“县县通高速”目标；汕昆、武深高速公路全线开工建设，河惠莞高速公路动工建设。完成赣深高铁“工可”评审。改造国省道465公里、县乡公路536公里，新建桥梁137座。新增公路里程1137公里，等级公路五年增加1375公里，增长10%；全市公路密度达102公里/百平方公里，五年增长9.1%。网络设施提档升级。积极推进“互联网+”，扎实推进信息化建设。实现网上办事大厅省、市、县、镇、村五级全覆盖。通信基础设施和信息化工程建设加快，2014年全市各大运营商宽带基础设施建设完成情况在全省名列前茅，2015年光纤入户率和4G基站建设完成率分别排名全省第一和第二。智慧城市建设扎实推进，江东新区成为国家智慧城市试点。水利设施提档升级。掀起民生水利建设新高潮，中小河流治理、病险水库除险加固、小流域综合治理、山洪灾害防治、城市防洪等水利防灾减灾工程和农田、民生水利工程建设取得明显成效。解决90万农村居民饮水不安全问题，灌区改造、小农水重点县建设、水利示范县建设、小农水示范镇建设等惠农项目成效显著。能源设施提档升级。五年完成电网建设资金28.8亿元，建成投运35千伏及以上项目31项，新增输电线路560千米、变电容量101.9万千伏安，供电能力进一步提高。

五、从生态看五年，始终守牢环保底线，绿色发展迈上新台阶。环保设施不断完善。新丰江水库成为国家重点支持的生态环境保护湖泊之一，总投资超15亿元的生态环境保护项目扎实推进。建成污水处理设施22座，日处理污水能力28.5万吨，配套管网232公里，五年分别增长150%、27%和32%。建成生活垃圾压缩中转站118个，自然村均设立垃圾收集点，县生活垃圾无害化填埋场全部建成使用。工业固体废物综合利用处置率达99.99%、医疗废物达标处置率达95%。监管力度不断加大。扎实推进南粤水更清行动计划，持续开展东江水环境综合整治。加强地质灾害防治。7个国土江河综合整治试点进展顺利。全面推进大气“五大治污”工程。深入开展清理禁养区养猪场和万绿湖非法网箱养鱼、治理东江水浮莲等专项行动，五年关闭养猪场1661家、餐饮场所38家、非法采矿点376个。全市划定乡镇集中式饮用水水源保护区98个。生态建设不断加强。成功创建“广东省林业生态市”。实施新一轮绿化河源大行动，积极推进“一灭三改”森林碳汇、生态景观林带、森林进城围城、乡村绿化美化等林业重点生态工程，森林覆盖率达74.3%。建成国家、省、市级生态示范村镇园区152个，五年增加67个。完成节能减排任务。

六、从文明看五年，创建工作成效斐然，社会事业迈上新台阶。教育创强成果显著。成功创建教育强市、6个教育强县（区）、100个教育强镇。促进学前教育优质健康发展，幼儿园从283所增加至536所，学前教育毛入园率提高到92.3％，五年提高21%。促进义务教育均衡发展，五年创建义务教育标准化学校483所，覆盖率达100%。市、县城区中小学校新建23所、改造扩建333所，新增学位7万多个，城市学位紧张状况大大缓解；其中市区新开办学校13所、改扩建学校39所，新增优质学位2.8万个。五县一区创建义务教育发展基本均衡县（区）通过国家验收。小学、初中、高中阶段学生毛入学率分别达100%、100%、93%。创建国家中等职业教育发展改革示范校3所，排名粤东西北地级市前列。新建扩建特殊学校6所，实现特殊教育学校县区全覆盖。教育质量提高，高考一本上线人数五年增长70%。成功举办第30届广东省青少年科技创新大赛。文化建设成果显著。高分获得全国文明城市提名资格。市图书馆、博物馆、文化馆、档案馆基本建成，河源恐龙博物馆、桃花水母大剧院等标志性文化设施建成使用，中心城区文化设施更加完善，文化活动更加活跃。全市文化馆站实现了100%达标，建成45个文化广场、100个乡镇（街道）文化站、1421个行政村（社区）文化室、1313家农家书屋。组建6个专业艺术表演团体。文化产业发展有新进展，巴伐利亚庄园、坚基演艺集团等大型骨干文化企业走在山区前列。创作了《红色河源》、《客家魂》和《桃花水母》等一批文艺精品。建成家训长廊、谚语长廊等一批文化传承教育基地，家训文化成为全国典型。打造了“多彩河源？欢乐广场”等群众文化活动品牌，举办了第二、三届客家文化节；编撰了《客家古邑文化书系》《河源市文化遗产汇编》等一批历史文化书籍；修缮了林寨、佗城、太平古街等一批历史文化遗址；挖掘保护了一批非物质文化遗产。市档案局被评为全国档案系统先进集体，市档案馆被评为国家一级档案馆。卫生计生工作成果显著。深化医药卫生体制改革，公立医院改革取得阶段性成效，基层医疗卫生机构历史债务全面化解。市人民医院二期工程、市妇幼保健院二期工程基本建成；市中医院二期工程、市公共卫生服务中心等项目建设扎实推进；新建和平县人民医院、龙川县第二人民医院、紫金县人民医院；完成66个卫生院标准化建设。市人民医院、中医院、妇幼保健院创建“三甲”医院进展顺利，医疗服务水平稳步提升。全市无重大传染病发生。坚持计划生育基本国策，较好完成省下达的人口计划。

七、从民生看五年，群众有更多获得感，保障水平迈上新台阶。社会保障体系进一步完善。建成101个镇街人力资源社会保障服务平台，五年新增城镇就业22.6万人、劳动力培训转移就业23.4万人、安置下岗失业人员再就业6.7万人，城镇登记失业率控制在2.45 %以内。城乡居民基础养老金标准提高到100元，居民医保财政补助标准提高到380元，五年分别提高82%、58%；参加城镇职工基本养老（含离退休）、城乡居民社会养老、城乡基本医疗、失业、工伤、生育保险人数五年分别增长96%、221倍、26%、19%、27%、66%；养老金水平人均五年增长68%。城镇、农村居民低保补贴水平分别提高到每人每月374元、172元以上，五年分别增长225%、187%。农村五保供养标准提高到当地上年度农村人均纯收入的60%以上，实现应保尽保。社会治理水平进一步提高。围绕打造“平安河源”，深入开展“3+2”等专项打击整治行动，五年破获各类刑事犯罪案件近2万宗，严重暴力犯罪案件破案率超过70%，有力震慑了违法犯罪。深入开展打假治劣工作，五年查处各类案件6500多宗，涉案货值达5亿多元，有效规范了市场经济秩序。全面完成“六五”普法规划，被评为省优秀；全市村（社区）法律顾问实现全覆盖；社区服刑人员特赦工作顺利完成。在全省率先开通网上信访大厅，信访维稳工作得到加强。推进社会创新项目建设，规范发展社会组织。安全生产、产品质量和食品药品监管、应急管理及处置等工作得到加强。脱贫攻坚步伐进一步加快。3年扶贫开发“双到”工作投入资金24亿元，283个重点村平均每村849万元。有劳动能力的贫困户人均纯收入9892元，比帮扶前增收7031元，脱贫率达100%。重点村村集体收入平均每村9.8万元，比帮扶前增收7万元。新修建村道789公里，新建或维修安全饮水工程249宗、卫生站212个、学校198个。完成农村低收入住房困难户住房改造12.44万户，“两不具备”村庄搬迁安置3.16万户。民生福祉进一步增进。坚持每年办好“十件实事”，五年民生支出676亿元，办好了50项110件群众看得见、摸得着、得实惠的实事，切实缓解上学、看病、交通、住房、饮水、就业、创业、低保等关系群众切身利益的突出问题。

此外，人事编制、民族宗教、社区建设、基层政权、社会组织、驻外机构、外事侨务、妇女儿童、人民武装、人民防空、防灾减灾、体育、优抚、双拥、科协、气象、打私、供销、老龄、残联、关工委、港澳台、殡葬等各项事业取得新成绩。

过去五年，政府系统扎实开展党的群众路线教育实践活动和“三严三实”专题教育活动，严格落实中央八项规定，坚决遏制“四风”，廉政建设进一步加强。顺利完成新一轮政府机构改革，推进行政审批标准化建设，推进商事制度和“三证合一、一照一码”登记制度改革，行政审批事项压减48.8%，行政审批事项全流程网上办理率大幅提升，政府效能进一步提高。认真开展“三赛”活动，建立“马上办”工作机制，破解一系列“卡壳”问题，服务发展水平进一步提升。主动接受监督，认真执行市人大及其常委会各项决议并向其报告工作，坚持向市政协通报情况，代表建议、政协提案办复率和基本满意率基本达100%。

各位代表！五年的实践，让我们深刻体会到：必须始终坚持加快发展不动摇，牢记发展第一要务，紧扣“三大抓手”，全力推动河源振兴发展，才能逐步摆脱贫困落后面貌；必须始终坚持绿富双赢不动摇，坚决守住生态底线，坚决实行源头控污，坚决强化末端治理，才能守住、巩固河源独特优势；必须始终坚持夯实基础不动摇，不以一时输赢论英雄，既重显绩更重潜绩，多做打基础谋长远工作，才能为长远发展铆足后劲；必须始终坚持改革创新不动摇，全面深化改革，大力实施创新驱动，努力推动转型升级，才能不断增添振兴发展新动力；必须始终坚持以人为本不动摇，铭记发展一切为了人民，切实改善民生，着力补齐短板，才能让广大人民群众有更多获得感；必须始终坚持务实创业不动摇，做到既着眼长远又立足当前，务实创业，狠抓执行，才能干一件成一件。

各位代表！过去五年，我市取得的成绩来之不易。这是省委、省政府和市委正确领导的结果，是全市人民团结拼搏、共同奋斗的结果，也离不开广州、深圳、中山等兄弟市和海内外各界人士的关心支持。在此，我代表市人民政府，向全市广大干部群众，向驻河源部队、消防官兵和各民主党派、人民团体、社会各界人士致以崇高的敬意！向长期关心支持我市改革发展的港澳同胞、台湾同胞、海外侨胞表示衷心的感谢！

在看到成绩的同时，我们也清醒地看到：一是我市总体处于发展的初级阶段，发展任务十分繁重，发展中不平衡、不协调的问题依然存在；二是我市经济规模不大，总体实力不强，支柱产业多为传统产业，处于价值链低端，创新发展不足，市场竞争力不强,战略性新兴产业还在培育之中，尚不足以对冲经济下行压力，推动经济从中高速向中高端转变压力巨大；三是城乡差距依然较大，中心城区辐射带动能力不强，农村基础设施薄弱，历史欠账多，脱贫攻坚任务艰巨；四是全面深化改革任重道远，城市管理体制仍未理顺，改革的力度仍需加大；五是社会事业短板较多，社会治理水平不高，基本公共服务均等化差距明显，保障和改善民生任务繁重；六是面对发展新常态，部分公务人员能力不适应，乱作为不作为在一些部门和单位仍有不同程度存在，有些部门工作氛围不浓，干劲不足。对这些问题，我们要高度重视，采取有力措施，认真加以解决。
